# Regulation of Pro-Apoptotic Phosphorylation of Kv2.1 K^+^ Channels

**DOI:** 10.1371/journal.pone.0129498

**Published:** 2015-06-26

**Authors:** Kai He, Meghan C. McCord, Karen A. Hartnett, Elias Aizenman

**Affiliations:** Department of Neurobiology, University of Pittsburgh School of Medicine, E1456 BST, 3500 Terrace St., Pittsburgh, PA, 15261, United States of America; Sackler Medical School, Tel Aviv University, ISRAEL

## Abstract

Caspase activity during apoptosis is inhibited by physiological concentrations of intracellular K^+^. To enable apoptosis in injured cortical and hippocampal neurons, cellular loss of this cation is facilitated by the insertion of Kv2.1 K^+^ channels into the plasma membrane via a Zn^2+^/CaMKII/SNARE-dependent process. Pro-apoptotic membrane insertion of Kv2.1 requires the dual phosphorylation of the channel by Src and p38 at cytoplasmic N- and C-terminal residues Y124 and S800, respectively. In this study, we investigate if these phosphorylation sites are mutually co-regulated, and whether putative N- and C-terminal interactions, possibly enabled by Kv2.1 intracellular cysteine residues C73 and C710, influence the phosphorylation process itself. Studies were performed with recombinant wild type and mutant Kv2.1 expressed in Chinese hamster ovary (CHO) cells. Using immunoprecipitated Kv2.1 protein and phospho-specific antibodies, we found that an intact Y124 is required for p38 phosphorylation of S800, and, importantly, that Src phosphorylation of Y124 facilitates the action of the p38 at the S800 residue. Moreover, the actions of Src on Kv2.1 are substantially decreased in the non-phosphorylatable S800A channel mutant. We also observed that mutations of either C73 or C710 residues decreased the p38 phosphorylation at S800 without influencing the actions of Src on tyrosine phosphorylation of Kv2.1. Surprisingly, however, apoptotic K^+^ currents were suppressed only in cells expressing the Kv2.1(C73A) mutant but not in those transfected with Kv2.1(C710A), suggesting a possible structural alteration in the C-terminal mutant that facilitates membrane insertion. These results show that intracellular N-terminal domains critically regulate phosphorylation of the C-terminal of Kv2.1, and *vice versa*, suggesting possible new avenues for modifying the apoptotic insertion of these channels during neurodegenerative processes.

## Introduction

Kv2.1, a voltage-gated K^+^ channel, plays diverse and important roles in neuronal function. This protein is highly expressed in mammalian brain tissue, including hippocampus, cerebral cortex, striatum, and thalamus [1, 2]. The Kv2.1 channel complex is comprised of four α subunits, each containing six membrane-spanning regions, a re-entrant P loop, and associated cytoplasmic N- and C-termini. Its channel activity is responsible for the majority of the delayed-rectifier K^+^ current in cortical, hippocampal, and sympathetic neurons [3–8]. Post-translational modifications of a relatively large number of amino acid residues in the intracellular domains of Kv2.1 are critical for regulating channel activity [9, 10]. Indeed, many residues undergo phosphorylation and dephosphorylation reactions in response to intracellular stress and extracellular stimulating signals [11, 12]. In most of these cases, changes in the overall phosphorylation status of Kv2.1, particularly at the C-terminus, are thought to mediate homeostatic, adaptive responses to alterations in neuronal activity or injurious stimuli [13–16]. There are, however, specific changes in the phosphorylation status of Kv2.1 at two unique amino acid residues, Y124 and S800, which are intimately linked to neuronal cell death processes.

Prior studies from our group have shown that oxidative and nitrosative injury promotes apoptosis in cortical neurons via the release of zinc from intracellular, metal-binding proteins [17–19]. The liberated zinc, in turn, activates p38 MAPK [20] and promotes Src kinase activity, leading to dual phosphorylation of Kv2.1 [21, 22]. This process is required for a Ca^2+^/CaMKII and SNARE-dependent membrane insertion of new Kv2.1 channels and the consequent increase in K^+^ currents necessary for cytoplasmic K^+^ loss during apoptosis [5, 23–25]. The phosphorylation of Kv2.1 by p38 MAPK occurs specifically at the S800 residue of the intracellular C-terminus [21], while Src mainly phosphorylates an N-terminal residue, Y124 [26]. Importantly, point mutations of either site to non-phosphorylatable amino acids (i.e. Y124F or S800A) eliminate the pro-apoptotic K^+^ current surge, and promote resistance to apoptotic injury [21, 22]. As such, mutated channels do not traffic to the plasma membrane following an apoptotic stimulus [21, 27]. This indicates that a coordinated phosphorylation-signaling pathway mediating p38 and Src kinases critically regulates Kv2.1 channels during oxidant-induced apoptosis [22, 28]. Still, whether phosphorylation events at S800 and Y124 occur independently of each other or are co-regulated via putative interactions of the N- and C-termini has not been investigated.

A complementary molecular mechanism of oxidant-triggered neuronal apoptotic cell death has been recently proposed, in which Kv2.1 channel oxidation of N-terminal C73 and C-terminal C710 residues can result in the formation of Kv2.1 oligomers and consequent cell death [29, 30]. These results, along with prior electrophysiological experiments, strongly suggest the existence of functional interactions between cytoplasmic N- and C-terminal domains of Kv2.1 [10, 31]. It is not known, however, how amino acids within different intracellular locations integrate signaling cascades, manifested as changes in phosphorylation status, to control channel activity.

In the present study, we investigate the molecular mechanisms regulating p38- and Src-dependent signaling cascades leading to pro-apoptotic Kv2.1 phosphorylation. Combining molecular, biochemical and electrophysiological techniques, we establish Src kinase as a critical upstream signaling modulator facilitating p38 MAPK phosphorylation of Kv2.1. Similarly, inability to phosphorylate the S800 residue negatively impact tyrosine phosphorylation of Kv2.1, likely at Y124. In addition, we show that mutations at Kv2.1 intracellular cysteine residues can dramatically affect pro-apoptotic phosphorylation of the channel, suggesting a critical, required interaction between cytoplasmic domains for the post-translational modification of pro-apoptotic residues.

## Materials and Methods

### Chemicals and reagents

Primary antibodies used in this study were from the following sources: mouse anti-Kv2.1 monoclonal antibody (clone K89/34) and rabbit anti-Kv2.1 antibody were obtained from NeuroMab (Davis, CA) and Alomone Lab (Jerusalem, Israel), respectively; mouse anti-phosphotyrosine monoclonal antibody (PY99) was from Santa Cruz (Dallas, TX), and mouse anti-GAPDH monoclonal antibody was from Novus (Littleton, CO). SB239063 was purchased from Tocris (Ellisville, MO). An affinity-purified rabbit phospho-specific antibody raised against Kv2.1 residue S800 was described previously [21]. Cell Extraction Buffer and NP40 Cell Lysis Buffer were from Invitrogen (Camarillo, CA). Bovine serum albumin (BSA) was from Jackson ImmunoResearch (West Grove, PA), and bicinchoninic acid (BCA) based protein assay reagents were from Thermo Scientific (Rockford, IL). Unless specified, all other chemicals used were of analytical grade quality or better and were from Sigma-Aldrich (St. Louis, MO).

### Plasmids

Wild-type Kv2.1, Kv2.1(WT), was a gift kindly provided by J. Trimmer (University of California, Davis, CA), Kv2.1(Y124F) was from A. Elson (Weizmann Institute, Rehovot, Israel). Kv2.1(C73A) and Kv2.1(C710A) were obtained from F. Sesti (Rutgers University, New Brunswick, NJ). Single amino acid substitutions, Kv2.1(S800A) and Kv2.1(S800E) were performed previously [21]. p38α and dominant negative p38 mutant (p38DN) were from J. Cavanaugh (Duquesne University, Pittsburgh, PA). Mouse c-Src plasmid (# 13663, deposited by J. Brugge, Harvard University, Cambridge, MA) was purchased from Addgene (Cambridge, MA).

### Cell culture, transfection, and protein sample preparation

Chinese hamster ovary (CHO) K1 cells (ATCC, Manassas, VA) were grown in Gibco Ham’s F12 nutrient medium (GlutaMax, Life Technologies, Grand Island, NY) with 10% fetal bovine serum (FBS) and 10 mM 2-[4-(2-hydroxyethyl) piperazin-1-yl] ethanesulfonic acid (HEPES). Cells were grown in 100 × 20 mm dishes and transfected when at 85% confluence using Lipofectamine (7.334 μg DNA in 55.1 μl Lipofectamine per dish) (Life Technologies, Grand Island, NY). To prepare total lysates for western blot experiments, we transfected cells with either wild type or mutant Kv2.1 DNA (10% of total DNA) and an appropriate amount of control empty vector, pcDNA3. For immunoprecipitation experiments, total DNA for transfection contained 10% wild type Kv2.1 or 30% mutant Kv2.1 plasmid DNAs (because of the low expression of mutant Kv2.1 proteins in CHO cells) as well as 15% of p38 or Src plasmid DNA plus appropriate amounts of control empty vector, pcDNA3. After 24 h, transfected CHO cells were utilized for electrophysiological recordings or briefly rinsed twice with ice-cold phosphate-buffered saline (PBS) and harvested for protein biochemistry. Cells were lysed in either Cell Extraction Buffer (for total protein detection) or NP40 Cell Lysis Buffer (for immunoprecipitation). Both were supplemented with complete protease inhibitor cocktail tablet (Roche, Penzberg, Germany) and freshly made phenylmethylsulfonyl fluoride (PMSF) according to the manufacturer’s instructions. Cellular debris in samples were removed by centrifugation at 10,000 × g for 10 min at 4°C. Protein samples were stored at -20°C and concentrations were measured using BCA protein assay kit.

### Immunoprecipitation and quantitative immunoblotting

Equal amounts (0.7–1 mg) of total protein from cell extracts were pre-cleared by adding 50 μl of resuspended volume of protein A/G plus-agarose (Santa Cruz, Dallas, TX) for 1 h at 4°C. After centrifugation, supernatants were mixed with mouse anti-Kv2.1 monoclonal antibody at 4°C for 3 h with gentle agitation before adding 90 μl of resuspended volume of beads overnight. The immune complex of protein-bound beads was washed and used in gel electrophoresis. Either beads or equal amount of total protein samples were incubated with 2× reducing sample buffer and heated at 100°C for 5 min before loading onto 8% gels. Sodium dodecyl sulfate-polyacrylamide gel electrophoresis (SDS-PAGE) was run using the Mini Protein 3 System (BioRad, Hercules, CA) and separated proteins were transferred onto 0.2 μm nitrocellulose membranes. The membranes were blocked with 1% BSA in PBS containing 0.05% Tween 20 (PBST) at room temperature for 1 h. In most experiments in this study, we took the advantage of Odyssey Infrared Imaging System (Li-Cor, Lincoln, NE) to detect two specific antigens of Kv2.1 simultaneously. To do so, two primary antibodies derived, respectively, from mouse and rabbit (mouse anti-Kv2.1 plus rabbit anti-phosphoKv2.1(S800) antibodies or mouse anti-phosphotyrosine plus rabbit anti-Kv2.1 antibodies) were diluted together in PBST and exposed to the blots. Later, after washing (3×) in PBST, membranes were incubated simultaneously with two Li-Cor IRDye-conjugated secondary antibodies raised specifically against mouse or rabbit. Either mouse or rabbit secondary antibody was labeled with IRDye 680LT (for 680 nm) or 800CW (for 780 nm). Different fluorescent signals from specific proteins were scanned and discriminated using two independent infrared detection channels at 685 and 785 nm excitation wavelengths and were quantified by the imaging system. Normalization procedures and other calculations, as well as statistical analyses are detailed in the figure legends.

### Electrophysiological measurements

Current recordings were performed at room temperature on eGFP-positive co-transfected CHO cells using the whole-cell patch clamp configuration technique as described previously [25]. The intracellular (electrode) solution contained (in mM): 100 K-gluconate, 11 EGTA, 10 KCl, 1 MgCl_2_, 1 CaCl_2_ x 2H_2_O, 10 HEPES; pH adjusted to 7.2 with concentrated KOH; 2.2 ATP and 0.33 GTP were added and the osmolarity was adjusted to 280 mOsm with sucrose. The extracellular solution contained (in mM): 115 NaCl, 2.5 KCl, 2.0 MgCl_2_, 10 HEPES, 10 D-glucose; pH adjusted to 7.2. Measurements were obtained with an Axopatch-1D amplifier and pClamp software (Molecular Devices, Sunnyvale, CA), using 2–3 MΩ recording electrodes. Electrodes were pulled from 1.5 mm borosilicate glass (Warner Instruments, Hamden, CT) with a model P-97 mechanical pipette puller (Sutter Instruments, Novato, CA). Series resistance was partially compensated (80%) in all cases. Currents were filtered at 2 kHz and digitized at 10 kHz with a Digidata 1440A (Molecular Devices). K^+^ currents were evoked with incremental 10 mV voltage steps to +80 mV from a holding potential of –80 mV. To determine current density values, steady-state current amplitudes were measured 180 msec after the initiation of the +10 mV step and normalized to cell capacitance.

### Statistical analysis

Normalized data are expressed as the mean ± S.E.M. and the level of significance was analyzed by one sample or pair-wise comparisons, or by one-way analysis of variance (ANOVA) with post hoc comparisons, as detailed in each figure legend.

## Results

To investigate the regulation of the phosphorylation events necessary for Kv2.1-facilitated apoptotic cascades, we employed the Chinese hamster ovary (CHO) cell expression system that we have previously characterized extensively [5, 27]. This model system has proven invaluable for the study of signaling pathways regulating Kv2.1 during cell death processes as it recreates the complete cascade found in neurons [5, 21, 25] and, importantly, lacks endogenous Kv2.1 channels [32], allowing for the use of channel phosphorylation mutants [21, 22, 28]. We began by performing a comparative assessment of wild type and various mutant Kv2.1 proteins expression profiles in CHO cells. Single point mutations at critical amino acids, namely Kv2.1(S800A) [21] and Kv2.1(Y124F) [22, 26], block the pro-apoptotic phosphorylation induced by p38 MAPK and Src kinase, respectively, while Kv2.1(C73A) and Kv2.1(C710A) prevent the formation of inter- or intra-subunit disulfide bridges within the channel previously proposed by Sesti and co-workers [29]. Cells were transfected with equal plasmid concentrations of either wild type (WT) or mutant Kv2.1 cDNA plasmids (S800A, Y124F, C73A, and C710A), and proteins were harvested 24 h later. Kv2.1(WT) protein was composed of multiple protein bands with a wide range of molecular weights (Panel A in S1 Fig). This was consistent with results obtained from native channels in neurons, as well in other cell types (e.g. COS-1 and HEK-293 cells) expressing recombinant Kv2.1 protein, indicating multiple phosphorylation profiles present on the channel protein, but not those generally associated with apoptotic events (i.e. not Y124 and S800) [11, 12, 33]. Moreover, when comparing overall mutant protein expression patterns, we observed that Kv2.1(C710A) protein primarily concentrated at higher molecular weight regions on gels, while Kv2.1(C73A), Kv2.1(S800A), and Kv2.1(Y124F) proteins displayed variable mobility patterns, but generally migrated to lower positions within the Kv2.1(WT) spectrum, especially Kv2.1(C73A). This suggests that the Kv2.1(C710A) mutant possesses electrophoretic mobility distinct from Kv2.1(C73A), Kv2.1(S800A) and Kv2.1(Y124F), could be indicative of a more highly phosphorylated protein [11, 12, 33], or some other process such as glycosylation [34]. Of note, when mutant Kv2.1 proteins were transiently expressed in CHO cells, the observable level of these mutant proteins was approximately 40% of Kv2.1(WT) (Panel B in S1 Fig). As such, single mutations on critical amino acids of Kv2.1 may affect channel expression levels and influence their overall electrophoretic migration on protein gels.

### Src influences p38-mediated phosphorylation of Kv2.1

**Fig 1:**
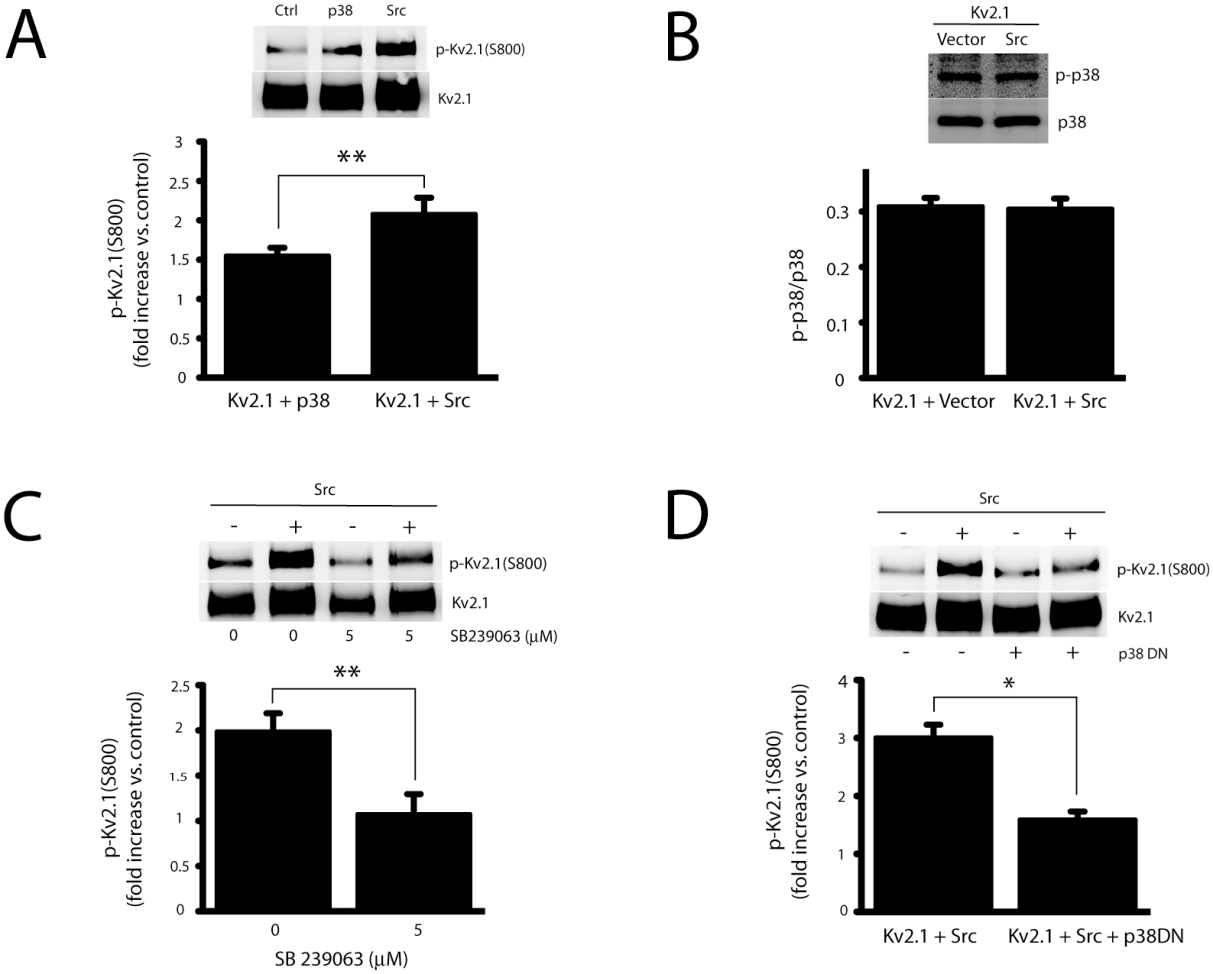
Src expression increases p38-dependent phosphorylation of Kv2.1 at S800. **A,** CHO cells were co-transfected with plasmid DNAs of Kv2.1 (10% of total DNA, see Methods), and either Src (15%) or p38 (15%). The membranes with immunoprecipitated Kv2.1 protein were co-probed with anti-Kv2.1 mouse monoclonal antibody and rabbit antibody specific against phosphorylation of Kv2.1 at residue S800. The levels of pKv2.1(S800) in Src- and p38-expressing CHO cells are expressed as the ratio of pKv2.1(S800) to total Kv2.1 protein, and normalized to the same ratio obtained from control cells (value of 1). The data represent mean ± SEM from 6–7 independent experiments (**p < 0.01, two-tailed, unpaired *t* test). **B,** Protein samples were harvested from Src- or control vector DNA-expressing CHO cells, the levels of total p38 protein (p38) and phosphorylated p38 protein (p-p38) in equal amounts of total cell lysates were detected by western blotting by using mouse antibody specific against p-p38 and rabbit antibody against total p38 protein. Results (mean ± SEM from 5 independent experiments) show that there is no change of p-p38 levels in Src-overexpressing CHO cells when compared with control cells. **C,** CHO cells were co-transfected with plasmid DNAs of Kv2.1 (10%), and either Src (15%) or control vector. Three hours later, transfected cells were treated with a specific p38 MAPK kinase inhibitor, SB 239063 (5 μM). Kv2.1 protein was immunoprecipitated and separated. Immunoblot was performed and quantified as described in Fig 1A. Values (mean ± SEM from 3 independent experiments) represent the ratios of the level of pKv2.1(S800) to total Kv2.1 normalized to their respective controls (-Src, no drug and—Src plus drug; **p < 0.01, two-tailed, paired *t* test). **D,** CHO cells were co-transfected with plasmid DNAs of Kv2.1 (10%) and either Src (15%), p38DN (15%) or control vector. Kv2.1 protein in transfected cells was immunoprecipitated, and quantified as described above. Values (mean ± SEM from 4 independent experiments) represent the ratio of the level of pKv2.1(S800) to total Kv2.1 normalized to respective controls, as in **C** (*p < 0.01, two-tailed, paired *t* test).

During apoptosis, Kv2.1 is a substrate of p38 and Src kinases; p38 MAPK mediates phosphorylation of Kv2.1 at residue S800 [21], while Src kinase targets residue Y124 [22, 26]. It has yet to be determined, however, whether there is an interdependence or co-regulation of the pro-apoptotic phosphorylation sites. To begin to explore this question, CHO cells were co-transfected with Kv2.1 as well as either Src or p38-expressing plasmids. Kv2.1 immunoprecipitates were probed with a rabbit antibody recognizing Kv2.1 only when it’s phosphorylated at residue S800 [21]. As expected [28], p38 co-expression significantly increased phosphorylation of Kv2.1(S800) (~1.6-fold over the level of basal phosphorylation; Fig 1A). Notably, Src expression also strongly stimulated Kv2.1(S800) phosphorylation (~2.2-fold increase over the level in control). To investigate whether Src overexpression in CHO cells enhanced p38 MAPK activity to induce the increased phosphorylation of Kv2.1(S800), we measured the level of phosphorylated (i.e. active) p38 in Src-expressing cells but noted no enhancement in the overall signal (Fig 1B). This indicates that Src over-expression, in and of itself, does not stimulate p38 activity, thereby suggesting that the increased phosphorylation of residue S800 in Kv2.1 was due to an alternative mechanism.

**Fig 2:**
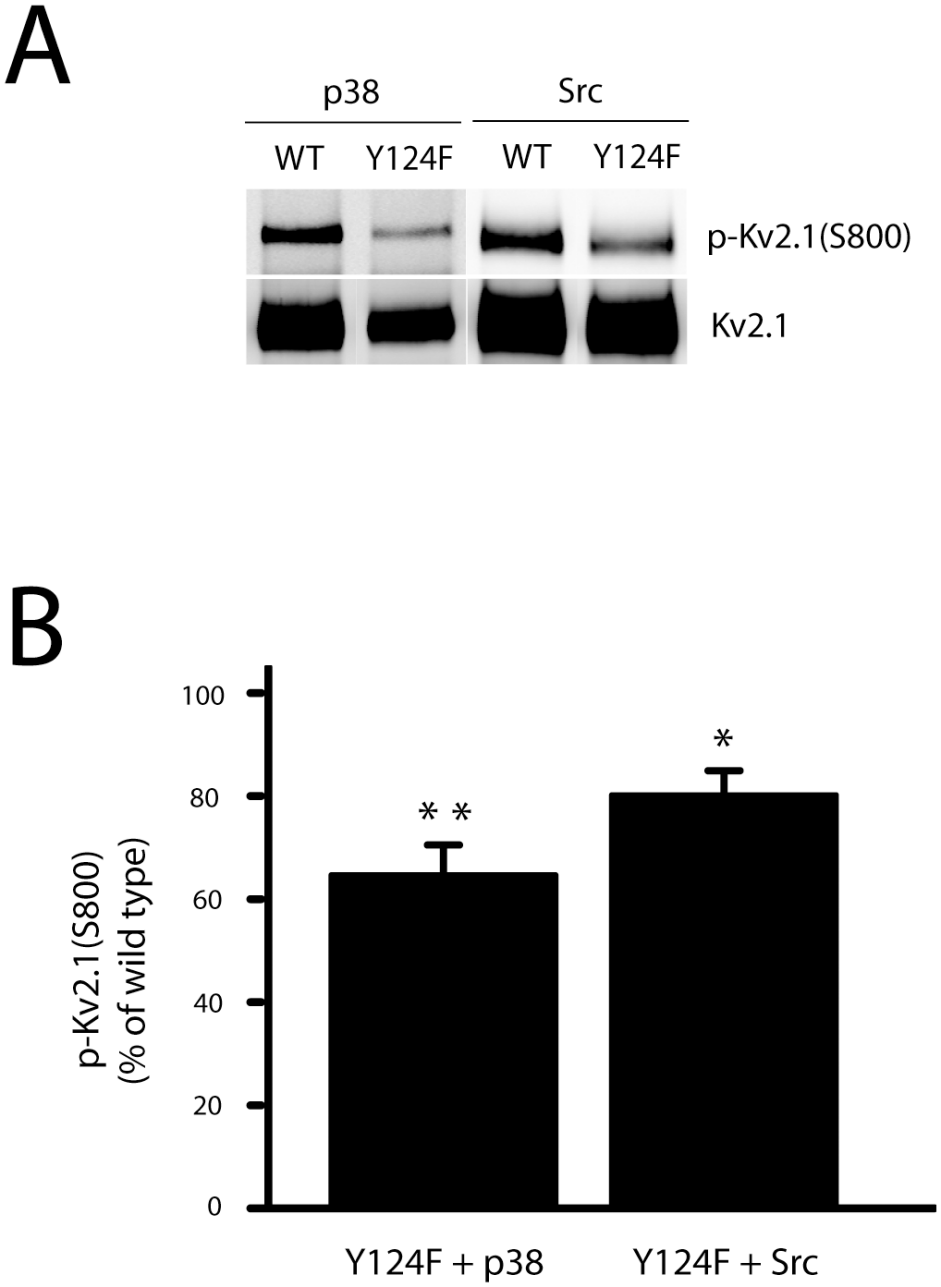
Kv2.1(Y124F) mutation blocks both Src- and p38-induced phosphorylation of Kv2.1 at S800. **A,** CHO cells were co-transfected with plasmid DNAs of Kv2.1 (10%) or Kv2.1(Y124F, 30%), and Src (15%) or p38 (15%). The membranes carrying with immunoprecipitated Kv2.1 protein complexes were co-probed with anti-Kv2.1 mouse monoclonal antibody and rabbit antibody specific against serine phosphorylation of Kv2.1 at S800, p-Kv2.1(S800). **B,** The signal densities of p-Kv2.1(S800) and total Kv2.1 proteins from Y124F mutants in either p38- or Src-expressing CHO cells (panels of Fig 2A) were quantified and the level of p-Kv2.1(S800) was expressed as a ratio of p-Kv2.1(S800) to total Kv2.1 protein and normalized to respective wild type controls (as 100%). The data represents mean ± SEM from 5 independent experiments for each condition (*p < 0.05 and **p < 0.01, one sample, two-tailed paired *t* test, vs. 100).

To confirm that p38 MAPK was indeed responsible for Src-stimulated phosphorylation of S800, we pre-treated cells with SB 239063 (5 μM), a selective p38 MAPK inhibitor [35]. We observed that SB 239063 almost completely blocked S800 phosphorylation of Kv2.1 induced by Src overexpression (Fig 1C). To further confirm this finding, we transfected a dominant negative p38 construct (p38DN) or its control vector into cells, together with Kv2.1(WT) and Src plasmids. This procedure also markedly reduced Kv2.1(S800) phosphorylation (Fig 1D). As there is some basal, endogenous p38 activity in the CHO cells (Fig 1B), and as also suggested by the presence of phospho-S800 signal under control conditions (see Fig 1A), these results indicate that p38-mediated phosphorylation of Kv2.1 at S800 is likely facilitated by the actions of Src, possibly via the phosphorylation of residue Y124 by this kinase. To evaluate this possibility, we utilized the mutant Kv2.1(Y124F), which lacks the main phosphorylation site for Src [26]. Using this mutant (Fig 2A), we observed a significantly decreased phosphorylation of the S800 residue both in p38- and Src-overexpressing CHO cells (Fig 2B). These results suggest that Src phosphorylation of Y124 positively regulates the phosphorylation of S800 on Kv2.1 by p38. It is noteworthy that in order to generate approximately similar levels of channel expression in these experiments (see S1 Fig), we employed 3 times as much Kv2.1(Y124)-expressing plasmid, when compared to Kv2.1 WT. A similar strategy was employed in all subsequent experiments utilizing Kv2.1 mutant plasmids.

### Mutant Kv2.1(S800A) specifically modifies Src effects on the channel

**Fig 3:**
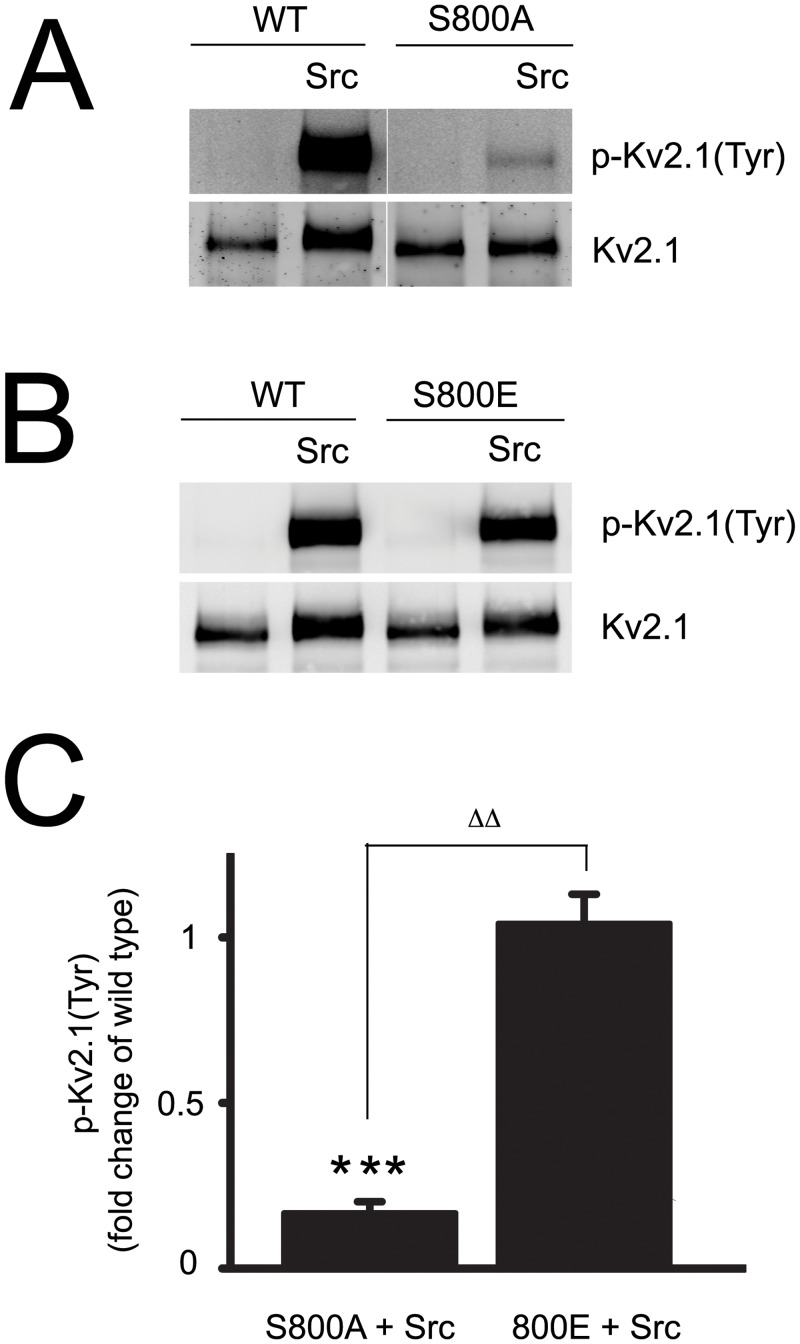
Src-induced tyrosine phosphorylation of Kv2.1 in CHO cells is significantly decreased in Kv2.1(S800A) mutants. **A,** CHO cells were co-transfected with plasmid DNAs of Kv2.1 (10%), Kv2.1(S800A) (10%), Src (15%) and vector controls. Protein samples were collected 24 h later and Kv2.1 protein was immunoprecipitated and transferred onto nitrocellulose membranes. The membranes were co-probed with mouse anti-phospho-tyrosine monoclonal antibody, p-Kv2.1(Tyr) and rabbit polyclonal antibody specific against total Kv2.1 (Kv2.1). **B,** CHO cells were co-transfected with plasmid DNAs of Kv2.1 (10%), Kv2.1(S800E) (10%), Src (15%) and vector controls, followed by experimental procedures described in Fig 3A. **C,** The signal densities of p-Kv2.1(Tyr) and total Kv2.1 proteins from Kv2.1WT, Kv2.1(S800A) and Kv2.1(S800E) were quantified as described earlier. The level of p-Kv2.1(Tyr) was expressed as a ratio of p-Kv2.1(Tyr) to total Kv2.1 protein and normalized to the tyrosine phosphorylation level of Kv2.1WT in CHO cells without Src overexpression. The data represents mean ± SEM from 4 independent experiments (***p < 0.001, compared with Kv2.1WT; one sample, two-tailed *t* test; and ^ΔΔ^ *p* < 0.01, two-tailed, paired *t* test).

Next, we investigated the reverse situation, namely, whether changes in the S800 site would affect Src-mediated phosphorylation of Kv2.1. Although we have not been able to generate successfully a phospho-specific antibody to residue Y124 [21], it has been previously demonstrated that a majority (>70%) of the phospho-tyrosine signal of immunoprecipitated Kv2.1 is derived from that residue [26]. These experiments were simplified by the fact that mutant Kv2.1(S800A) channels are insensitive to p38 phosphorylation, while Kv2.1(S800E) effectively mimics the p38-phosphorylated state [22]. In contrast to the previously noted basal p38 phosphorylation of residue S800 (Fig 1A), CHO cells appear not to show resting phospho-tyrosine activity, at least with regards to immunoprecipitated Kv2.1 protein, nor does co-expression of p38 increase phospho-tyrosine signal (not shown). However, co-expression of Src induced a remarkable increase in tyrosine phosphorylation of wild-type Kv2.1 (Fig 3A and 3B). The levels of Src-induced phospho-tyrosine activity were significantly reduced in Kv2.1(S800A) mutant channels (Fig 3A), while phospho-tyrosine levels in Kv2.1(S800E) channels were comparable to wild type (Fig 3B). These data suggest that Src-related tyrosine phosphorylation of Kv2.1 can be modulated by p38-mediated phosphorylation at S800, again pointing to mutual regulatory activities at the pro-apoptotic phosphorylation sites on Kv2.1.

### Cysteine mutations influence p38 phosphorylation of Kv2.1

**Fig 4:**
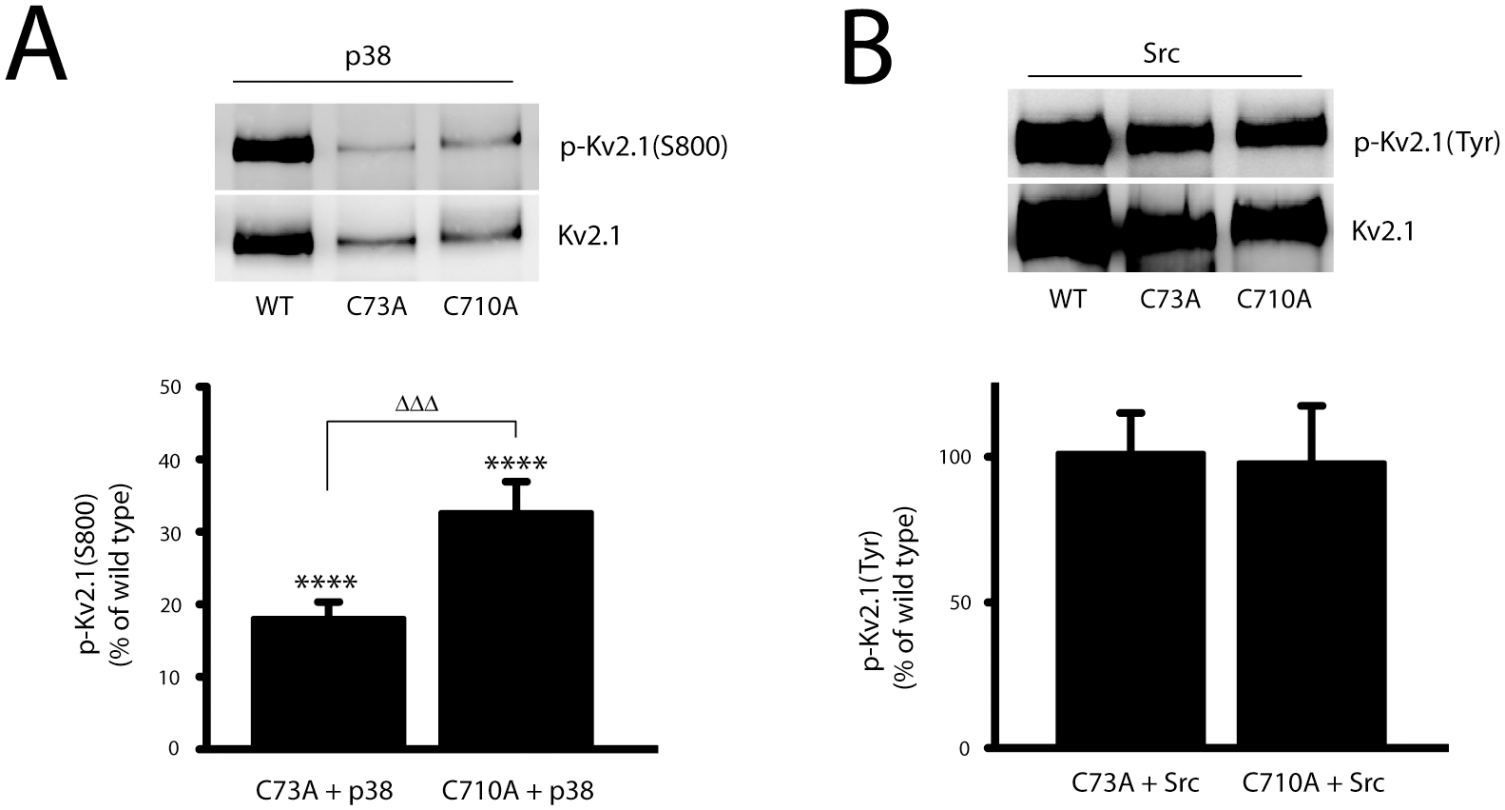
N- and C-terminal cysteine residues differentially influence Kv2.1 phosphorylation. **A,** CHO cells were co-transfected with plasmid DNAs of p38 (15%), and Kv2.1(WT) (10%), Kv2.1(C73A) (30%), or Kv2.1(C710A) (30%). The membranes with separated immunoprecipitated Kv2.1 protein complexes were co-probed with mouse anti-Kv2.1 monoclonal antibody and rabbit polyclonal antibody specific against serine phosphorylation of Kv2.1 at S800, p-Kv2.1(S800). The level of p-Kv2.1(S800) was calculated from the ratio of p-Kv2.1(S800) to total Kv2.1 protein, and then normalized to the level of p-Kv2.1(S800, WT) in p38-transfected CHO cells. The values represent mean ± SEM from 7 independent experiments (****p < 0.0001, compared with Kv2.1WT; one sample, two-tailed *t* test; and ^ΔΔΔ^ *p* < 0.001, two-tailed paired *t* test). **B,** CHO cells were co-transfected with plasmid DNAs of Src (15%), and Kv2.1 (WT, 10%), Kv2.1(C73A, 30%), or Kv2.1(C710A, 30%). Immunoblot was co-probed with rabbit anti-Kv2.1 polyclonal antibody (Kv2.1) and mouse anti-phosphotyrosine antibody, p-Kv2.1(Tyr). The signal densities of p-Kv2.1(Tyr) and total Kv2.1 proteins from Kv2.1WT, Kv2.1(C73A) and Kv2.1(C710A) were quantified as described above. The level of p-Kv2.1(Tyr) was calculated as the ratio of pKv2.1(Tyr) to total Kv2.1 protein and normalized to tyrosine phosphorylation of Kv2.1WT in CHO cells with Src overexpression. Similar p-Kv2.1(Tyr) levels were detected in Src-expressing CHO cells in WT, C73A and C710A groups.

A recent study showed that a cysteine residue (C73) located in the N-terminus of Kv2.1 is involved in the process of neuronal apoptosis associated with oxidation of the channel. Indeed, mutating C73 to an alanine was sufficient to induce cytoprotection [29]. Furthermore, C73 was proposed by Sesti and co-workers to interact with cysteine 710 (C710) of the Kv2.1 C-terminus through an inter- or intra-subunit disulfide bond [29]. As such, we tested whether C73 and C710 may influence the phosphorylation of pro-apoptotic residues of Kv2.1. First, we compared the effects of p38-induced S800 phosphorylation in wild type and cysteine mutant channels. We observed that phosphorylation of S800 by p38 co-expression was substantially reduced in CHO cells transfected with either Kv2.1(C73A) or Kv2.1(710A), the effects being markedly more pronounced in the C73A mutant (Fig 4A). In contrast, tyrosine phosphorylation of Kv2.1 induced by Src co-expression in the cysteine mutant channels was relatively comparable to Kv2.1(WT) (Fig 4B). These results suggest that mutations at cysteine residues that promote putative interactions between the N and C termini of Kv2.1 can have pronounced effects on p38, but not Src phosphorylation of the channel.

**Fig 5:**
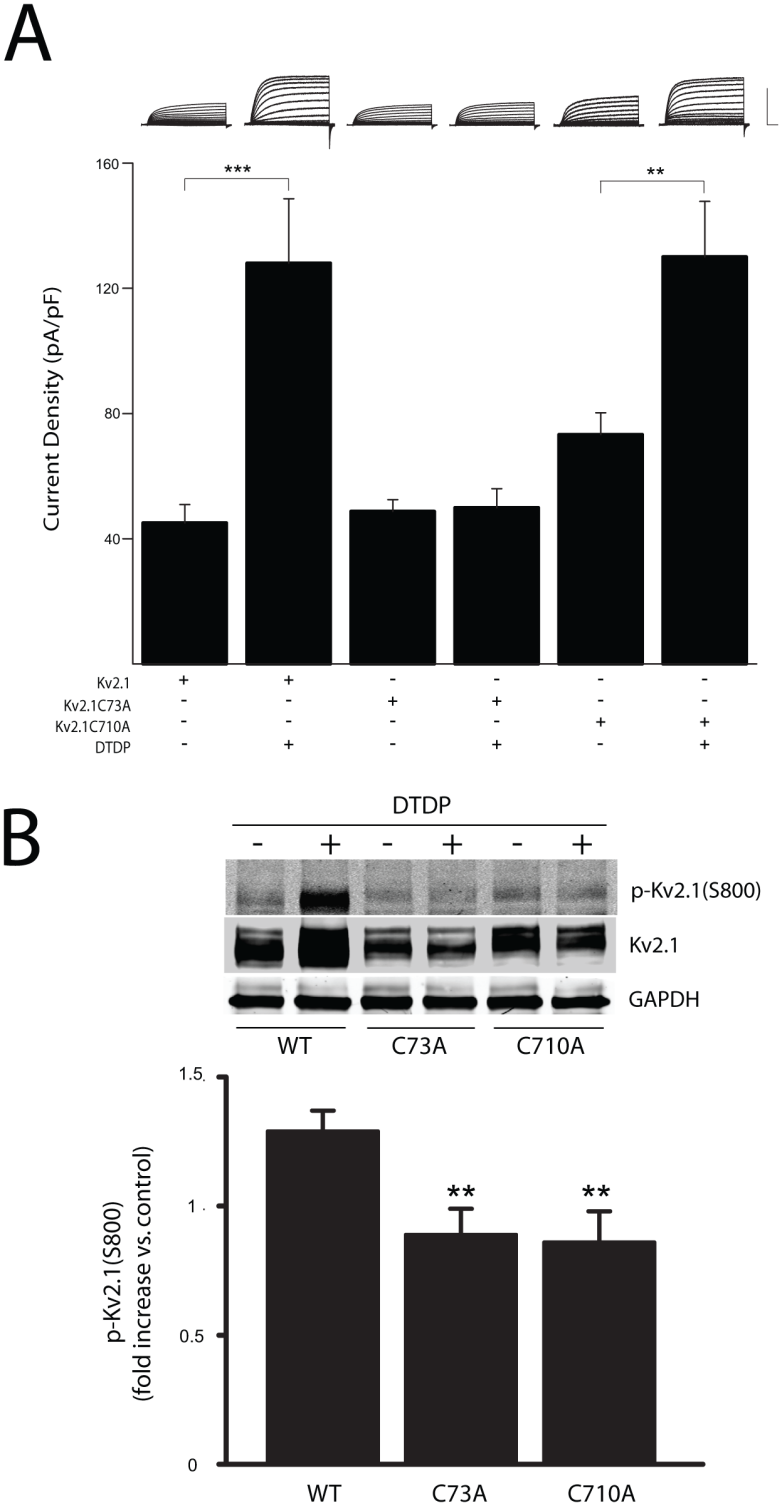
(A) **Suppression of apoptotic current enhancement in Kv2.1(C73A)- but not Kv2.1(C710A)-expressing K** ^**+**^ **channels.** Representative whole-cell K^+^ currents and pooled mean ± SEM current densities recorded from Kv2.1-expressing CHO cells without (n = 10) or with (n = 6) 30 μM DTDP, Kv2.1C73A-expressing CHO cells without (n = 9) or with (n = 7) 30 μM DTDP, or Kv2.1C710A-expressing CHO cells without (n = 9) or with (n = 12) 30 μM DTDP. Following DTDP exposure, cells were maintained in the presence of the broad spectrum protease inhibitor 1-3-Boc-aspartyl(Ome)-fluoromethyl ketone (BAF; 10 μM)-containing medium to enhance viability and facilitate electrophysiological recordings [21]. Results show that expression of the C73A channel mutant, but not the C710A mutant, prevents the increase in Kv2.1-mediated K^+^ currents triggered by DTDP. Currents were evoked by a series voltage steps from -80 mV to +80 mV, in 10 mV increments. To determine current density values, steady-state current amplitudes were measured 180 msec after the initiation of the +10 mV step and normalized to cell capacitance. Scale bars: 5 nA, 25 msec; **p<0.01, ***p<0.001, ANOVA/Bonferroni. (B) Kv2.1WT- and mutants-transfected CHO cells were treated with 30 μM DTDP for 10 min and incubated BAF (10 μM)-containing culture medium for 30–60 min. Total lysates were collected and ran for western blots (top). The membranes were co-probed with Kv2.1 and p-Kv2.1(S800) antibodies. GAPDH protein was used as a loading control. The levels of p-Kv2.1(S800) were calculated from the ratio of p-Kv2.1(S800) to total Kv2.1 protein, and then normalized to the levels of p-Kv2.1(S800) in vehicle-treated controls, respectively (bottom). DTDP-induced phosphorylation of both cysteine mutants was inhibited, when compared to wild type Kv2.1 channels. The values represent mean ± SEM from 4 independent experiments (**p < 0.01, ANOVA/Dunnett).

In previous studies [21, 22], we reported that the phosphorylation of Kv2.1 by Src and p38 kinases is associated with oxidant-triggered apoptotic K^+^ current surges in transfected CHO cells. Moreover, mutation of either the Y124 or S800 into non-phosphorylatable residues was sufficient to prevent Kv2.1 current enhancement following oxidant treatment. Moreover, using an alkylatable, extracellular cysteine-containing mutant channel (I379C), we were also able to demonstrate that a serine to alanine mutation at residue S800 prevented the membrane insertion of Kv2.1 following injury [22]. Since mutations at C73 or C710 could differentially prevent p38 phosphorylation of the S800 residues, we evaluated whether CHO cells expressing the C73A and C710A cysteine mutant channels would also fail to manifest an apoptotic K^+^ current surge. CHO cells expressing either wild type Kv2.1 or either Kv2.1(C73A) or Kv2.1(C710A) were exposed to a 10 minute treatment with the thiol oxidizing agent 2,2’-dithiodipyridine (DTDP; 30 μM), a treatment that results in a pronounced increase in K^+^ currents approximately 3 hours post-treatment [5, 20]. As expected, Kv2.1-expressing cells showed enhanced whole-cell K^+^ currents following DTDP exposure (Fig 5A). In contrast, CHO cells transfected with Kv2.1(C73A) mutant channels failed to express an increase in K^+^ currents, even though basal currents were comparable to those observed in WT-expressing cells (Fig 5A). This finding was consistent with the limited phosphorylation by p38 observed in this mutant (Fig 4A). Surprisingly, cells expressing Kv2.1(C710A) channels behaved similarly to WT channels. As such, we further compared the level of DTDP-induced phosphorylated S800 in CHO cells transfected with wild type Kv2.1 to S800 phosphorylation levels in cells expressing either Kv2.1(C73A) or Kv2.1(C710A). Here, we found that in both mutant Kv2.1-expressing cells, the amount S800 phosphorylation following DTDP exposure was completely blocked, when compared to wild-type expressing cells (Fig 5B). These results are in line with the results shown in Fig 4A, but cannot account for the lack of DTDP-induced current enhancement observed in the Kv2.1(C710A) mutant. It is possible that this mutation alters the conformation of the channel sufficiently to promote membrane insertion following Y124 phosphorylation but without a concomitant S800 phosphorylation. Future studies will address this possibility.

## Discussion

A zinc-triggered cellular signaling cascade resulting in enhanced K^+^ currents and cellular K^+^ loss is critical for promoting oxidant-mediated neuronal cell death [36]. This process is mediated by both Src kinase-induced N-terminal Y124 phosphorylation and p38 MAPK-mediated C-terminal S800 phosphorylation of the Kv2.1 delayed rectifier K^+^ channel [21, 22]. The regulation of pro-apoptotic Kv2.1 channel phosphorylation processes by coordinated Src and p38 kinase activity, however, had heretofore not been explored. In this study, using variants of recombinant Kv2.1 proteins expressed in CHO cells, we report that phosphorylation events at Y124 and S800 are tightly co-dependent, and seem to be strongly influenced by putative N- and C-terminal interactions, previously proposed by Sesti and co-workers to be mediated by critical intracellular cysteine residues C73 and C710 [29], likely as a result of previously reported pro-oxidant sulfhydryl residue modification of these amino acids [29, 30]. Although there is a gap in our knowledge regarding the structure of the very long cytoplasmic intracellular tail that is unique to Kv2 channels [37, 38], FRET-based studies do suggest that during channel activation, segments of the N-terminal so-called T1 domain, which encompasses C73, can come in close apposition to the C-terminal activation (CTA) domain, which is a bit downstream of C710, but it does encompass S800 (CTA: residues 741–853) [39]. Importantly, truncation and immunoprecipitation based studies Mohapatra and Trimmer [31] strongly argue that there is a physical interaction between segments of the C-terminus and the N-terminus of Kv2.1.

Especially notable in our studies is a requisite intact N-terminal Y124 for successful p38 phosphorylation of C-terminal S800, as well as a promotion of the phosphorylating actions of the MAPK with increasing Src activity. These results provide support to the idea that the overall regulation of Kv2.1 function is highly dependent of N- and C-terminal interactions under both physiological [31], and, as shown here and in prior studies [29, 30], pathophysiological conditions.

Although the dramatic influence of the Y124F mutation on S800 phosphorylation strongly points to the aforementioned scenario, the enhanced activity of p38 MAPK in Src-overexpressing cells could also have been the result of a direct upstream action of the tyrosine kinase on p38 function. Indeed, in murine Kupffer cells, it has been reported that Src family kinases can regulate p38 MAPK-mediated production of cytokine interleukin-6 (IL-6) following hypoxia [40]. In addition, Src family kinase-dependent p38 MAPK activation is suggested in the involvement in the signaling pathway of epidermal growth factor (EGF)-stimulated intestinal epithelial cell migration [41]. In our studies, however, we found no changes in the levels of background-phosphorylated p38 when cells overexpressed Src, a condition that nonetheless leads to enhanced p38-mediated phosphorylation of Kv2.1 residue S800. These results strongly suggest that the actions of Src on Y124 are permissive for enhanced phosphorylation of the C-terminal S800 residue by p38.

Fyn is a Src family tyrosine kinase that has been shown to physically associate with Kv2.1 protein in mouse Schwann cells [42]. Moreover, focal adhesion kinase (FAK) can also bind to Kv2.1, a process that promotes activation of the kinase in neuronal and non-neuronal cells [43]. In an unrelated study, we reported that inhibition of Src family kinases can lead to FAK dysfunction in developing cortical neurons in cultures [44], suggesting that these two kinases can be functionally interrelated under certain conditions. As such, it is possible that Kv2.1 channels act as a scaffolding proteins to harbor Src family kinases so that the channel structure/function can be tightly regulated [45]. Because Src-induced tyrosine phosphorylation of Kv2.1 is substantially diminished in mutant Kv2.1(S800A)-expressing CHO cells, but not in Kv2.1(S800E)-transfected CHO cells, this may be indicative of a structural change of Kv2.1 channel protein in Kv2.1(S800A) mutant that disfavors tyrosine phosphorylation, a condition which we have established to be associated with the inhibition of apoptogen-induced membrane insertion of Kv2.1 [21, 28]. Whether p38 is also part of this putative signaling complex remains to be established and will be the subject of a future study.

In conclusion, we have demonstrated that Src protein kinase strongly promotes serine phosphorylation of Kv2.1 channel at p38-targeted residue S800 via a process independent of enhanced cellular p38 MAPK activity. Our cysteine mutant experiments also suggest that a single cysteine mutation, either at C73 or C710 of the Kv2.1 channel, significantly alters the state of S800 phosphorylation. As such, the difference in S800 phosphorylation present in these mutants could account, at least in part, for the previously described anti-apoptotic properties of the Kv2.1(C73A) mutant observed in transfected cells following oxidative stress [29, 30].

## Supporting Information

S1 Fig Mutant Kv2.1 proteins expressed in CHO cells demonstrate varying electrophoretic mobility. **A,** CHO cells were co-transfected with 10% plasmid DNA of wild type (WT; of total DNA, See Methods) or each mutant Kv2.1 (C73A, C710A, S800A, Y124F) plus 90% empty vector DNA. Protein samples were collected 24 h later, and equal amounts of protein from cell lysates were separated by 8% reducing SDS-PAGE and transferred onto nitrocellulose membranes. The levels of total Kv2.1 proteins were detected by western blotting by using mouse antibody specific against non-phosphorylated Kv2.1; GAPDH protein was also probed with a specific antibody and used as internal protein loading controls. No Kv2.1 protein was detected when vector DNAs were expressed in CHO cells. **B,** Quantification of mutant Kv2.1 expression in CHO cells as shown as the ratio calculated from total Kv2.1 to GAPDH, and then normalized to Kv2.1(WT) expression (100%, as indicated by dash line). The data are expressed as mean ± SEM from three independent experiments; all mutant Kv2.1 protein expression is significantly lower than Kv2.1(WT) protein in transfected CHO cells (*p* < 0.05 or 0.01, compared to Kv2.1(WT); one sample, two-tailed *t* test).
(PDF)
