# Supplementary material for: Regulation of Pro-Apoptotic Phosphorylation of Kv2.1 K+ Channels
Source: PLoS One. 2015 Jun 26;10(6):e0129498. doi: 10.1371/journal.pone.0129498 (PMC4482604; doi:10.1371/journal.pone.0129498)
Supplement: S1 Fig — A, CHO cells were co-transfected with 10% plasmid DNA of wild type (WT; of total DNA, See Methods) or each mutant Kv2.1 (C73A, C710A, S800A, Y124F) plus 90% empty vector DNA. Protein samples were collected 24 h later, and equal amounts of protein from cell lysates were separated by 8% reducing SDS-PAGE and transferred onto nitrocellulose membranes. The levels of total Kv2.1 proteins were detected by western blotting by using mouse antibody specific against non-phosphorylated Kv2.1; GAPDH protein was also probed with a specific antibody and used as internal protein loading controls. No Kv2.1 protein was detected when vector DNAs were expressed in CHO cells. B, Quantification of mutant Kv2.1 expression in CHO cells as shown as the ratio calculated from total Kv2.1 to GAPDH, and then normalized to Kv2.1(WT) expression (100%, as indicated by dash line). The data are expressed as mean ± SEM from three independent experiments; all mutant Kv2.1 protein expression is significantly lower than Kv2.1(WT) protein in transfected CHO cells (p < 0.05 or 0.01, compared to Kv2.1(WT); one sample, two-tailed t test). (PDF) [file pone.0129498.s001.pdf]

Supporting Information

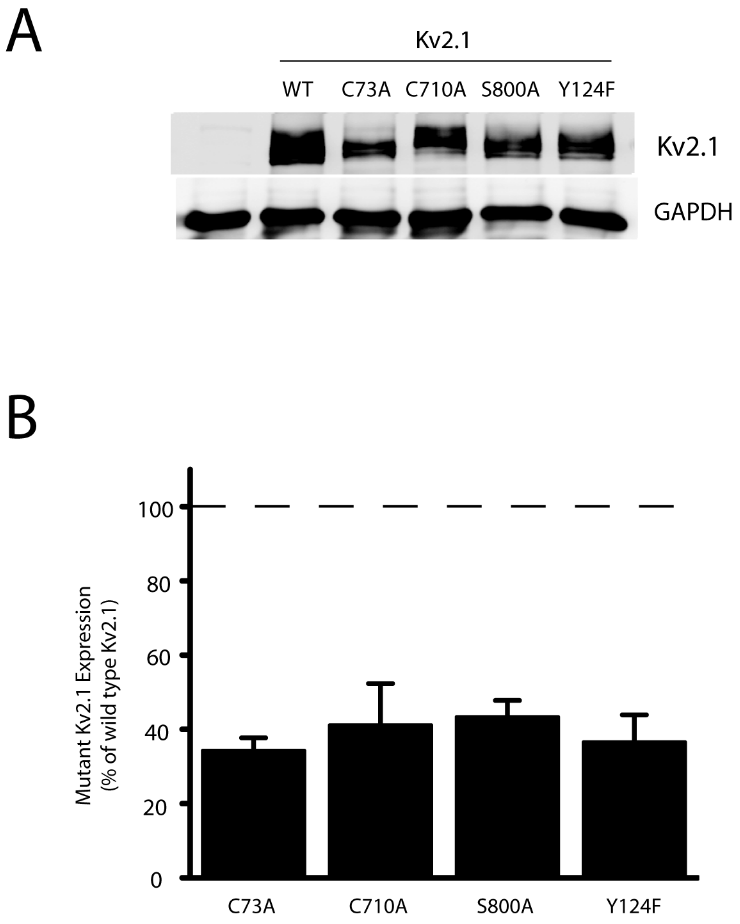

S1 Fig. Mutant Kv2.1 proteins expressed in CHO cells demonstrate varying electrophoretic mobility. A, CHO cells were co-transfected with 10% plasmid DNA of wild type (WT; of total DNA, See Methods) or each mutant Kv2.1 (C73A, C710A, S800A, Y124F) plus 90% empty vector DNA. Protein samples were collected 24 h later, and equal amounts of protein from cell lysates were separated by 8% reducing SDS-PAGE and transferred onto nitrocellulose membranes. The levels of total Kv2.1 proteins were detected by western blotting by using mouse antibody specific against non-phosphorylated Kv2.1; GAPDH protein was also probed with a specific antibody and used as internal protein loading controls. No Kv2.1 protein was detected when vector DNAs were expressed in CHO cells. **B,** Quantification of mutant Kv2.1 expression in CHO cells as shown as the ratio calculated from total Kv2.1 to GAPDH, and then normalized to Kv2.1(WT) expression (100%, as indicated by dash line). The data are expressed as mean ± SEM from three independent experiments; all mutant Kv2.1 protein expression is significantly lower than Kv2.1(WT) protein in transfected CHO cells ($p < 0.05$ or 0.01, compared to Kv2.1(WT); one sample, two-tailed t test).
